# Acceptability and Feasibility of Online Support Groups for Mental Health Promotion in Brazilian Graduate Students During the COVID-19 Pandemic: Longitudinal Observational Study

**DOI:** 10.2196/44887

**Published:** 2023-10-13

**Authors:** Aneliana da Silva Prado, Elisabeth Kohls, Sabrina Baldofski, Christine Rummel-Kluge, Joanneliese de Lucas Freitas

**Affiliations:** 1 Department of Psychiatry and Psychotherapy, Medical Faculty Leipzig University Leipzig Germany; 2 Department of Psychology Federal University of Parana Curitiba Brazil; 3 Campus Curitiba Federal Institute of Education, Science and Technology of Parana Curitiba Brazil; 4 Department of Psychiatry and Psychotherapy, University Leipzig Medical Center Leipzig University Leipzig Germany

**Keywords:** support group, online group, COVID-19 pandemic, higher education, graduate students, university students, mental health, online intervention, internet intervention, e–mental health, mental health promotion, feasibility, students, acceptability

## Abstract

**Background:**

The outbreak of the COVID-19 pandemic in 2020 aggravated already existing difficulties and added new challenges for students. Owing to the gap between needed and available psychological services, group interventions may offer a helpful strategy for student mental health promotion.

**Objective:**

This study aimed to investigate the acceptability and feasibility of a 4-week online support group program designed for mental health promotion tailored to graduate students at a Brazilian public university in the context of the COVID-19 pandemic (May 2022 to June 2022).

**Methods:**

Participants in the program took part in online support groups based on a pilot group facilitated by a trained clinical psychologist. Self-administered, standardized web-based questionnaires were assessed at the baseline (T0; before the intervention), postintervention (T2), and follow-up (T3; after 4-6 weeks) time points. We measured sociodemographic variables, treatment credibility and expectancy (Credibility and Expectancy Questionnaire), satisfaction (Client Satisfaction Questionnaire), negative effects of the intervention (Negative Effects Questionnaire), depressive symptoms (Patient Health Questionnaire–9 [PHQ-9]), and participants’ quality of life (abbreviated World Health Organization Quality of Life assessment). A 9–answer option questionnaire and open-ended questions also assessed the group’s perceived positive and negative outcomes.

**Results:**

The total sample comprised 32 participants. Most (23/32, 72%) were doctoral students. Credibility and expectancy scores were high. Participants’ satisfaction (Client Satisfaction Questionnaire) with the program was high at the postintervention (T2) and follow-up (T3) evaluations (T2: mean 28.66, SD 3.02; T3: mean 27.91, SD 3.02). Most participants reported that they could learn from other participants’ experiences (T2: 29/32, 91%; T3: 27/32, 84%) and felt encouraged to take better care of themselves (T2: 22/32, 69%; T3: 24/32, 75%). None of the participants reported that they had no benefits from the program. The PHQ-9 scores showed mild to moderate depressive symptoms (mean 9.59, SD 6.34), whereas the answers of 9% (3/32) of the participants to the PHQ-9 item 9 indicated suicidality at baseline (T0). Finally, the 4 domains of quality of life (physical: *P*=.01; psychological: *P*=.004; social: *P*=.02; and environmental: *P*<.001) showed a slight and statistically significant improvement at the postintervention evaluation (T0: mean 57.03, SD 15.39 to 59.64, SD 17.21; T2: mean 64.32, SD 11.97 to 68.75, SD 8.87).

**Conclusions:**

Online support groups for the mental health promotion of graduate students are feasible and can be especially useful for universities with students allocated to different cities. They are also satisfactory and may positively influence participants’ quality of life. Therefore, they can be considered a helpful mental health promotion strategy in the educational context. Further studies could evaluate these (or similar) programs under nonpandemic circumstances.

## Introduction

### Background

In recent years, studies have reported an increase in graduate students’ mental health issues [1-10]. Specifically, symptoms of anxiety, depression, loneliness, and low self-efficacy have been reported, resulting in difficulties coping with academic tasks and writing dissertations or theses and even in discontinuation of studies [11-15].

The outbreak of the COVID-19 pandemic in 2020 not only aggravated already existing difficulties but also added new challenges for students. Students’ socioeconomic conditions and the availability of social support were negatively affected. Moreover, the shift to web-based study courses; the necessary changes or postponements of research projects; and the infrastructure problems regarding internet access, equipment, library access, or a suitable home environment to study in—to name just a few burdens—presented a very challenging situation for students [16-20]. During the pandemic, alcohol abuse, stress, grief, anger, anxiety, and depressive symptoms significantly increased among university students worldwide [16,18,21-28]. Therefore, the call for mental health promotion programs tailored to this target group became even more compelling after the onset of the pandemic [16,22].

There are many challenges that graduate students in particular face [2,8,10,11]. First, they must develop their research project more autonomously compared with the structured organization they experienced in a bachelor or undergraduate course, which requires a higher level of responsibility, organization, and time management. Second, writing a dissertation or thesis can be a very challenging task for some students. Third, graduate students tend to have less social support from peers than they receive in undergraduate courses. Finally, especially in Brazil, graduate students need to work while studying because of serious financial difficulties (eg, only a few scholarships available and rising inflation) [12,14]. Owing to these specific challenges, graduate students are even more at risk of developing mental health issues [11,29]. Therefore, universities need to provide proactive care strategies for graduate students.

Nevertheless, the services offered by universities are usually insufficient to reach all students who need psychosocial support. The increased demand for counseling services in universities is higher than the currently available psychological services and counseling supply [2,5,12,13,19,25,29-31]. The Brazilian public mental health system is organized through the Psychosocial Care Network (*Rede de Atenção Psicossocial* in Portuguese), comprising primary care, specialized mental health care, crisis management services, inpatient units, deinstitutionalization initiatives, and psychosocial rehabilitation programs [32]. It is an underfunded area within the Brazilian Unified Health System [33] with significant gaps in the provision of services in some regions that rely only on primary care in addition to the structural and social barriers to accessing mental health services, such as stigma, discrimination, economic disparities, and racism [32].

In this context, group interventions may offer a good alternative [34-36]. Considering the stigma associated with mental health problems, group interventions that are not diagnosis specific but rather target broader aspects of students’ issues and, thus, aim at primary prevention may offer a helpful strategy for student mental health promotion [13,36-38]. An integrative literature review of studies implementing (face-to-face) group interventions for university student mental health promotion and prevention conducted by Souza et al [37] showed the effectiveness of most group interventions on mental health promotion and prevention and better cost-effectiveness than individual counseling. Furthermore, it has been pointed out that group interventions aimed at promoting mental health in university students may improve mental health outcomes [37], self-care, and care about others [31,36]; empower participants [34]; decrease loneliness; increase the feeling of being part of a group or community [39]; and encourage learning from others’ experiences [35]. Furthermore, studies have shown that an increased sense of belonging to the university community and increased empowerment might decrease the rates of discontinuation of studies [10,12].

A systematic review of web-based mental health promotion and prevention interventions for youth aged 12 to 25 years conducted by Clarke et al [38] indicates the potential of these interventions to promote youth well-being and reduce mental health issues. They pointed out that participant support in web-based interventions is a relevant factor of these interventions regarding completion and outcomes. Nevertheless, the authors recommended further research on participants’ engagement with and disengagement from web-based interventions regarding expectations, motivation, personality, experiences, and preferences.

Internet interventions for mental health interventions may vary in type, approach, and definition [40,41]. Web-based synchronous interventions are those in which the interaction takes place simultaneously between the people involved in real time, as is the case with phone or video calls, for example [40-42]. During the pandemic, the use of various types of internet interventions increased, boosting eHealth [40]. Owing to social distancing measures and the shift to web-based activities, guided synchronous web-based mental health promotion group interventions targeting university students in Brazil were developed [31,39]. To date, no study has evaluated online support groups tailored to Brazilian graduate students. In addition, regarding web-based group therapy, studies on video groups are rare, and web-based group intervention research is in its infancy [39,40].

### Objectives

Considering the challenges that graduate students have been facing in the pandemic context and the potential benefits of online support groups, this study aimed to investigate the acceptability and feasibility of a 4-week online support group program for mental health promotion tailored to graduate students of a Brazilian public university in the COVID-19 pandemic context. The intervention was offered as a mental health promotion strategy to improve individuals’ coping strategies and quality of life rather than ameliorating symptoms or deficits, as stated by the World Health Organization [43]. User satisfaction and the perceived positive and negative outcomes were also assessed. We hypothesized that online support groups would be feasible and acceptable as a mental health promotion strategy. Furthermore, it was hypothesized that this could increase the quality of life.

## Methods

### Context of the Study

This study was conducted at the Federal University of Paraná (UFPR), a large Brazilian public university. In 2022, the university had 6949 graduate students enrolled; of these, 656 (9.44%) were professional master’s students, 2984 (42.94%) were academic master’s students, and 3309 (47.62%) were doctoral students [44].

When the intervention evaluated in this study was conducted (May 1, 2022, to June 30, 2022), the percentage of the population that was fully vaccinated against COVID-19 had risen from 76.51% to 78.82%. The reproduction rate of COVID-19 cases in this period was 1.01 on the first day and 1.20 on the last [45,46]. The UFPR was the first Brazilian federal university to resume face-to-face teaching on February 14, 2022 [47].

### Participants and Recruitment

**Figure 1:**
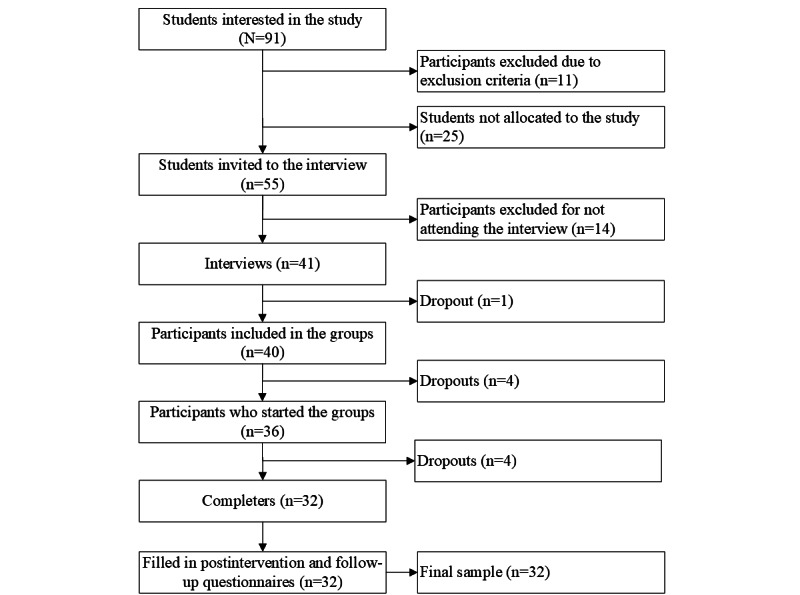
Flowchart of the recruitment, selection, and composition procedures for the sample.

All the graduate students at the UFPR, Brazil, were invited via email to participate in a 4-week online support group program on mental health promotion. After subscribing and answering screening questions on the inclusion and exclusion criteria, the students received an email to be interviewed by a trained clinical psychologist via web (the first author [AdSP]; Figure 1). In the interview, participants received information about the group and the study procedures (15-30 min).

The inclusion criteria were being enrolled as a graduate student, being aged ≥18 years, having an adequate understanding of the Portuguese language, and having internet access. The exclusion criteria were having a thesis or dissertation planned to be finalized before the follow-up questionnaire, having a previous psychosis diagnosis, and not attending the interview. The psychosis diagnosis was screened through a self-report item in the web-based subscription form and further in the interview through 2 screening questions from the Mini-International Neuropsychiatric Interview [48]. The sample was naturalistic and not diagnosis specific.

There were 4 groups intended to have 6 to 10 participants each. On the basis of subscription order, participants were allocated to each group based on their participation availability. A total of 41 students were interviewed and eligible for participation in the study. This number is because 1 participant dropped out before the groups started and a student from the waiting list filled in their spot. There were 4 participants who missed the first session and 4 who missed at least 2 sessions; therefore, 32 participants were considered completers and were included in the final sample. This study considered dropout if participants did not answer the baseline questionnaire, missed the first group session, or missed ≥2 of the following sessions. The students who were excluded from the study because of the exclusion criteria (n=11), could not be allocated to the study (n=25), or did not attend the scheduled interview (n=14) were informed by email that they could not take part in the study and received information about other support services provided by the UFPR and the Brazilian public health system.

### Pilot Group and Development of the Online Group Intervention

The design of the online support group was based on a pilot group of 4 sessions of approximately 90 minutes carried out in March 2021 with 6 female students aged between 24 and 46 years.

The pilot group had a leading question for each session based on the participants’ ideas presented during an initial baseline interview. From the leading question, the participants responded and interacted with each other and with the facilitator and cofacilitator. In each session, coping strategies regarding the participants’ issues were discussed, as well as the participants’ strengths and how they had helped themselves [49-51]. The principles of safety and dignity were observed.

The first session aimed to provide participants with brief information about the group’s purpose, confidentiality, and voluntary participation. The topic of this session was the impact of the pandemic on their experiences as graduate students. The second session’s topic was time management. It aimed to discuss and develop participants’ strategies to increase self-efficacy in organizing their studies. The third session had the interpersonal relationships with advisors and peers as the main topic, and the participants discussed their difficulties in this area and came up with ideas to improve their relationships with advisors and peers. Finally, in the fourth and last session, the participants talked about the meaning of graduation in their life and their expectations, goals, and values, rethinking their potential to solve some of the problems they identified. They also identified vulnerabilities and difficulties in a graduate student’s learning and development process.

The participants were interviewed via web before and once again after the pilot group finished (open interviews). Both interviews were video recorded and later transcribed by 3 undergraduate students trained by the researchers (AdSP and JdLF). The transcriptions were analyzed qualitatively through content analysis as presented by Bardin [52] and were then used to design the intervention presented in this study. Content analysis is one of the most common methods used for analyzing qualitative data [53], with Bardin [52] as one of the main references in Brazil [53]. Content analysis is performed through coding, which is a transformation of the raw data of the text through cutting, aggregating, and enumerating according to precise rules, which allows for the expression of content that can serve as indexes [52].

The qualitative analysis of the transcribed interviews indicated that participants viewed the online support group favorably. They expressed that the group provided social support and empowerment regarding graduate students’ issues, and they expressed a desire to stay connected with one another when the group was over. Participants also suggested that the online support group should have more sessions and be offered as a permanent program regardless of the pandemic context. Furthermore, they pointed out internet connection problems and concerns about the privacy of information regarding their relationship issues with their advisors as the participants were mostly in the same graduate program.

Overall, the pilot group results suggested the potential for online support groups to play a positive role in providing graduate students with social support, coping strategies, belongingness, and mental health awareness. In addition to the facilitator’s moderation, peer support was a distinctive feature of the pilot group. All participants’ cameras were on during the sessions—unless they had an internet connection problem and needed to turn them off—to have a better connection with and commitment to the group. None of the participants dropped out of the pilot group.

### Online Support Groups

The online support group program was intended to promote mental health, enhance students’ strengths, and offer peer support through active listening. On the basis of the Psychological First Aid (PFA) principles [49-51,54], the program was intended neither as a long-term solution nor as psychotherapeutic treatment or psychological counseling but to provide psychosocial support and encourage self-care and coping strategies for the difficulties addressed by graduate students. According to PFA principles, it was intended to be a simple, nonintrusive method of care that involves active listening to support calmness during times of crisis to protect a person from further harm, including psychological distress [49,51,54]. During the COVID-19 pandemic, support groups and PFA web-based interactions were recommended to enhance coping and connectedness [51].

The intervention consisted of 4 weekly online synchronous sessions held in 4 groups of 6 to 9 participants each, with 2 groups having the sessions in May 2022 and 2 having them in June 2022. The weekly sessions took place at a fixed time and lasted for 90 minutes each. A trained clinical psychologist (AdSP) conducted the interviews and moderated the sessions and the pilot group. She is a master’s-level psychologist experienced in administering online groups and psychoeducation. The UFPR provided the software for the sessions, so the students were already familiar with it. Participants were informed that they could already know other participants from the university context as it was a naturalistic sample. As video and audio were used, the group was not held anonymously, and the sessions were recorded (all participants provided informed consent before study participation).

There was a central topic for each session, which was presented as a question at the beginning. The topics of the sessions were based on the pilot group and included the following: (1) perceived burdens of the pandemic on the graduate experience, (2) time management, (3) interpersonal relationships with advisors and peers, and (4) self-care strategies.

The facilitator moderated the exchanges between participants regarding their experiences and coping strategies. If necessary, the facilitator slightly adapted the topic discussed during the session to the participants’ suggestions or mentioned needs. In case of severe crises (eg, reporting self-injury or suicidal ideation), the facilitator had a standard operating procedure adapted to the remote situation.

### Measures

#### Overview

Intervention outcomes were measured through self-report questionnaires filled in using the web-based tool Enterprise Feedback Suite Survey (version 21.1; UNIPARK) in Portuguese. Assessments were conducted at the baseline (T0; before the intervention), postintervention (T2), and follow-up (T3; after 4 weeks; Table 1) time points.

**Table 1:** Measures used in the study.

Instrument	Time point		
	T0	T2	T3
Sociodemographic information	✓		
CEQ^a^ (adapted)	✓	✓	✓
Expectation (1 item)	✓		
CSQ-8^b^		✓	✓
Satisfaction (1 item)		✓	✓
Positive outcomes from the support group (1 item)		✓	✓
Use of other support services		✓	✓
NEQ^c^ (adapted)		✓	✓
Negative aspects (1 item; open-ended)		✓	✓
PHQ-9^d^	✓		
WHOQOL-BREF^e^	✓	✓	✓

^a^CEQ: Credibility and Expectancy Questionnaire.

^b^CSQ-8: Client Satisfaction Questionnaire–8.

^c^NEQ: Negative Effects Questionnaire.

^d^PHQ-9: Patient Health Questionnaire–9.

^e^WHOQOL-BREF: abbreviated World Health Organization Quality of Life assessment.

Feasibility and acceptance were assessed via participants’ satisfaction measured using the Client Satisfaction Questionnaire–8 (CSQ-8); participants’ credibility and expectancy were measured using the Credibility and Expectancy Questionnaire (CEQ); and positive and negative outcomes were measured using a 9–answer option questionnaire (“how did you benefit as a graduate student from participating in the online support group?”), the Negative Effects Questionnaire (NEQ), and open-ended questions at the postintervention (T2) and follow-up (T3) time points. Furthermore, participants’ quality of life was measured using the abbreviated World Health Organization Quality of Life assessment (WHOQOL-BREF).

#### Sociodemographic Measures

Questions related to sociodemographic and academic information (age, gender, sexual orientation, course level, faculty, income, change in income, residential situation, relationship status, being a parent, and chronic physical conditions) were assessed. Moreover, the presence of previously diagnosed mental disorders, as well as previous psychotherapeutic treatment for these mental disorders, was investigated.

#### Treatment Credibility and Expectancy

The CEQ [55] was used to measure treatment credibility at the baseline (T0), postintervention (T2), and follow-up (T3) time points. The researchers translated the CEQ using a back translation procedure and slightly adapted the wording to the intervention format. The CEQ includes a credibility and an expectancy factor—each factor has 3 items. For the credibility factor, all items were measured on a rating scale from 1 to 9. The total sum scores of the credibility and expectancy factors ranged from 3 to 27—higher scores indicated higher credibility or expectancy. The questionnaire has demonstrated high test-retest reliability and adequate internal consistency [55].

As an additional question to the CEQ, at baseline (T0), participants were asked how much they agreed with the following statement—“I believe that an online support group for graduate students, mediate by a moderator, could help me”—on a 4-point Likert scale (1=*strongly agree*, 2=*agree*, 3=*disagree*, and 4=*strongly disagree*). Furthermore, at the postintervention (T2) and follow-up (T3) evaluations, participants were asked how much they agreed with the following sentence—“I found it easier to address topics that were unpleasant to me in the online support group than in direct personal contact with other students”—on a 4-point Likert scale ranging from 1 to 4 (1=*it applies completely*, 2=*it applies*, 3=*it doesn’t apply*, and 4=*it doesn’t apply at all*).

#### Satisfaction

The CSQ-8 [56] was used to measure participants’ satisfaction with the group at the postintervention (T2) and follow-up (T3) evaluations. The CSQ-8 was translated using a back translation procedure and compared with an available Portuguese version [57], and the wording was adapted slightly to the intervention format. All CSQ-8 items were measured on 4-point Likert scales. The total sum score of the questionnaire ranged from 8 to 32—higher scores indicated higher satisfaction. The reliability of the CSQ-8 is generally high, and its internal consistency is sufficient [56].

#### Positive and Negative Outcomes of the Online Support Groups

In the postintervention (T2) and follow-up (T3) questionnaires, participants were asked about how they benefited from participating in the online support group (eg, feeling listened to and supported by the other participants and learning from others’ experiences) as well as about the negative aspects of the online support group and suggestions they had to improve the program. The following question—“how did you benefit as a graduate student from participating in the online support group?”—had 9 answer options that participants could choose and an open-ended question to explain in case they chose “other.”

Moreover, there were two open-ended questions related to this outcome: (1) “Is there any negative aspect you would like to point out about the online support group?” and (2) “Are there any suggestions you would like to do to improve the program?” As participants used the final open-ended question to make comments about the positive and negative outcomes of the online support groups that they felt were not included in the previous answers, this final open-ended question was also included in this analysis.

#### Use of Other Support Services

In the postintervention (T2) and follow-up (T3) questionnaires, participants were asked which other supportive offers they had used in the last 2 weeks other than the online support group sessions (eg, social contacts, psychotherapist, offers from the mental health services at the university, and social media) in a 5-item questionnaire and an open-ended option to point out other services.

#### Negative Effects

Potential negative outcomes were measured using the short version (20 items) of the NEQ [58,59], which investigates the occurrence and characteristics of negative effects of psychological treatments on 6 derived factors: symptoms, quality, dependency, stigma, hopelessness, and failure. The NEQ was translated using a back translation procedure, and the wording was adapted slightly to the intervention format and used for the postintervention (T2) and follow-up (T3) evaluations. The questionnaire consists of a 4-point Likert scale (0=*not at all*; 4=*extremely*) and differentiates between negative effects attributed to psychological treatment and those possibly caused by other circumstances, providing a mean score of the negative impact of the intervention. Overall, the results are presented as total frequencies, means, and SDs for the full measure (for negative effects related to treatment). The questionnaire also includes 1 open-ended question to capture other negative effects that are not included in the items. The NEQ was found to have good internal consistency [59].

#### Depressive Symptoms

The Patient Health Questionnaire–9 (PHQ-9) [60,61] was used to assess depressive symptoms over the last 14 days, measured using 9 items on a 4-point Likert scale (0=*not at all*; 3=*nearly every day*). The sum score ranges from 0 to 27, with higher scores indicating higher levels of depressive symptoms. The internal consistency of the PHQ-9 is high [60].

#### Quality of Life

The WHOQOL-BREF [62,63] was used to assess participants’ quality of life at the baseline (T0), postintervention (T2), and follow-up (T3) time points. The 26 items of the questionnaire were rated on a 5-point Likert scale (1=*not at all*; 5=*extremely*). In total, 4 different domains of quality of life were evaluated: physical, psychological, social, and environmental. An index was calculated for each domain ranging from 0 to 100, with higher index scores representing a higher quality of life. The WHOQOL-BREF has demonstrated good discriminant validity, content validity, test-retest reliability, and internal consistency [62,63].

### Statistical Analysis

All analyses were conducted using SPSS Statistics (version 27.0; IBM Corp). The significance level applied to statistical testing was Cronbach α=.05 (2-tailed). For the NEQ, after variable value transformation in SPSS, the scoring matrix (Excel [Microsoft Corp] spreadsheet) provided by the authors [64] was used to build the NEQ score results.

First, descriptive analyses were conducted for sociodemographic and academic variables, participation rates, treatment expectancy and credibility, participants’ satisfaction, positive outcomes, negative effects, depressive symptoms, and quality of life. In this paper, income is presented in real (the Brazilian currency), whose symbol is “R$.” When this paper was written, a currency exchange rate of R$ 1=US $0.19 was applicable.

Second, participants’ satisfaction (CSQ-8) was evaluated at the postintervention (T2) and follow-up (T3) time points. Wilcoxon tests (nonparametric paired groups) were administered as the tests for normal distribution showed nonnormally distributed values (Shapiro-Wilks tests; *P*<.05). All effect sizes were interpreted as suggested by Cohen [65], meaning that effect sizes between 0.21 and 0.39 were considered small, effect sizes between 0.40 and 0.79 were considered medium, and effect sizes of ≥0.80 were considered large.

Third, positive and negative outcomes and the open-ended question on the NEQ were evaluated using qualitative analysis [66].

Finally, potential differences in quality of life (WHOQOL-BREF) and treatment credibility and expectancy (CEQ) between the different time points (baseline [T0], postintervention [T2], and follow-up [T3]) were tested using repeated-measure ANOVA. A Greenhouse-Geisser correction was applied to correct for the lack of sphericity in the repeated-measure ANOVA when applicable. The Bonferroni correction was applied to correct for multiple testing when applicable.

A post hoc power analysis using the G*Power software (version 3.1.7; Heinrich-Heine-Universität Düsseldorf) [67] was conducted with the following specifications: quality of life as the outcome variable and small effect size with 2-tailed α=.05 for a repeated-measure ANOVA with one group, with a sample of 40 participants. The results revealed that a power of 78.2% was obtained under these conditions.

### Ethical Considerations

This study was approved by the ethics committee of the UFPR (5.337.739) and registered in “Plataforma Brasil” (Certificate of Presentation for Ethical Consideration: 39593120.0.0000.0102). The participants provided web-based opt-in informed consent to take part in this study.

## Results

### Sample Characteristics

The total sample comprised 32 participants. The only variable that showed a statistically significant difference between dropouts and completers was course level (χ^2^_1_=4.5; *P*=.03; master’s students: 6/15, 40% dropouts and 9/15, 60% completers; doctoral students: 3/26, 11% dropouts and 23/26, 88% completers). There were no statistically significant differences between dropouts and completers regarding age (*P*=.18), being a parent (*P*=.64), relationship status (*P*=.43), residential status (*P*=.82), course year (*P*=.07), income before the pandemic (*P*=.91), or current income (*P*=.52).

The participants were aged between 22 and 49 years (mean age 32.63, SD 7.32 y), and most of them identified as female (25/32, 78%) and heterosexual (24/32, 75%). Most participants lived with other people (eg, partners, children, or roommates; 26/32, 81%) and did not have children (27/32, 84%). Of the 5 participants who had children, 4 (80%) reported that they had children aged <18 years—multiple answers were possible. Regarding relationship status, 59% (19/32) were married or in a relationship, and 41% (13/32) were single or divorced.

Regarding their studies, 72% (23/32) of the participants were doctoral students, and 28% (9/32) were master’s students. In total, 56% (18/32) were enrolled in their first or second year of the graduate course, and 44% (14/32) were in their third year or above. A total of 10 different faculties were represented in the sample: agricultural sciences (5/32, 16%), biology sciences (5/32, 16%), law sciences (5/32, 16%), human sciences (5/32, 16%), applied social sciences (3/32, 9%), hard sciences (3/32, 9%), health sciences (2/32, 6%), technological sector (2/32, 6%), education (1/32, 3%), and geographical sciences (1/32, 3%).

Regarding income, 41% (13/32) of the participants reported having a per capita income range of R$ 1651 to R$ 3300 (approximately US $315-US $630) before March 2020, whereas 66% (21/32) of the participants reported earning this income range currently. Furthermore, 56% (18/32) of the participants indicated that their income had not changed since March 2020, when the COVID-19 pandemic started, whereas 31% (10/32) indicated that their income had decreased and 12% (4/32) indicated that their income had increased. The main reasons for the income increase were receiving a scholarship to complete the graduate course (3/4, 75%), finding a new job (1/4, 25%), and moving back to live with their parents (1/4, 25%). The main reasons for an income decrease were inflation (5/10, 50%), scholarships that had not been adjusted to inflation rates (2/10, 20%), unemployment (2/10, 20%), reduction in working hours (1/10, 8%), quitting their job to complete the graduate course (1/10, 8%), moving house (1/10, 8%), and health problems (1/10, 8%); multiple answers were possible.

The reported sources of income were a scholarship (23/32, 72%), family support (8/32, 25%), full-time jobs (4/32, 13%), part-time jobs (2/32, 6%), self-employment (2/32, 6%), and other (2/32, 6%; multiple answers were possible).

Regarding mental disorders, 31% (10/32) of the participants reported that they had been diagnosed with a mental disorder. Specifically, depression (7/32, 22%), anxiety disorder (6/32, 19%), and obsessive-compulsive disorder (1/32, 3%) were reported by the participants (multiple answers were possible). There were 6% (2/32) of participants who reported having an autism spectrum disorder diagnosis. Participants were also asked about current psychotherapy treatment: 25% (8/32) were doing web-based therapy, and 3% (1/32) were doing face-to-face therapy. Finally, 16% (5/32) of the participants reported having a chronic physical condition.

### Participation

The completion rates for each group were as follows: 90% (9/10) for group 1, a total of 90% (9/10) for group 2, a total of 60% (6/10) for group 3, and 80% (8/10) for group 4. Throughout all groups, the session in which there was more absenteeism of participants was the third one, which was missed by 12% (4/32) of the participants, followed by the second session, which 9% (3/32) of the participants missed, and the last session, which was missed by 3% (1/32) of the participants. In all these cases, participants voluntarily informed the researcher about the reasons why they could not join the session.

### Treatment Credibility and Expectancy

The credibility (mean 24.19, SD 2.5) and expectancy (mean 21.09, SD 3.5) scores were high in the baseline evaluation. As an additional question to the CEQ, 53% (17/32) of the participants agreed and 47% (15/32) of the participants strongly agreed with the following statement: “I believe that an online support group for graduate students, mediated by a moderator, could help me.”

In the postintervention (T2) evaluation, both scores increased (credibility: mean 25.66, SD 1.5; expectancy: mean 22.38, SD 5.0; Table 2). Furthermore, 50% (16/32) of the participants agreed and 31% (10/32) of the participants strongly agreed with the following statement: “I found it easier to address topics that were unpleasant to me in the online support group than in direct personal contact with other students.”

**Table 2:** Results of assessments at the baseline (T0), postintervention (T2), and follow-up (T3) time points and test values (n=32).

Variable		T0	T2	T3	Test value
**Treatment credibility and expectancy (CEQ^a,b^), mean (SD)**					
	Credibility factor	24.19 (2.5)^c^	25.66 (1.5)^d^	24.34 (3.1)^c^	*F*_1.400,43.386_=4.386; *P*=.03; η^2^=0.124
	Expectancy factor	21.09 (3.5)^c^	22.38 (5.0)^c^	21.00 (4.7)^c^	*F*_1.546,47.926_=2.239; *P*=.13; η^2^=0.067
Satisfaction (CSQ-8^e^), mean (SD)		N/A^f^	28.66 (3.02)^c^	27.91 (3.02)^d^	*Z*=−2.055; *P*=.04; *r*=0.36
Depressive symptoms (PHQ-9^g^), mean (SD)		9.59 (6.34)	N/A	N/A	N/A
**Quality of life (WHOQOL-BREF^h,i^), mean (SD)**					
	Physical	58.26 (13.80)^c^	65.96 (12.42)^d^	64.17 (14.30)^c,d^	*F*_2,62_=4.988; *P*=.01; η^2^=0.139
	Psychological	57.03 (15.39)^c^	64.32 (11.97)^d^	62.11 (13.15)^c,d^	*F*_2,62_=6.163; *P*=.004; η^2^=0.166
	Social	59.64 (17.21)^c^	64.49 (16.22)^d^	64.58 (14.97)^c,d^	*F*_2,62_=4.141; *P*=.02; η^2^=0.118
	Environment	59.18 (11.56)^c^	68.75 (8.87)^d^	66.60 (11.62)^d^	*F*_2,62_=22.395; *P*<.001; η^2^=0.419

^a^Credibility: T0-T2: *P*=.02, Cohen *d*=.54; T2-T3: *P*=.009, Cohen *d*=.60; T0-T3: *P*>.99, Cohen *d*=.04. Expectancy: because no statistically significant difference was found between the periods of assessment, no post hoc analysis was applied.

^b^CEQ: Credibility and Expectancy Questionnaire.

^c^Indicates the statistically significant differences in the scores between the periods of assessment based on post hoc analysis (Bonferroni post hoc).

^d^Indicates the statistically significant differences in the scores between the periods of assessment based on post hoc analysis (Bonferroni post hoc).

^e^CSQ-8: Client Satisfaction Questionnaire–8.

^f^N/A: not applicable.

^g^PHQ-9: Patient Health Questionnaire–9.

^h^WHOQOL-BREF: abbreviated World Health Organization Quality of Life assessment.

^i^Physical: T0-T2: *P*=.009, Cohen *d*=.62; T2-T3: *P*>.99, Cohen *d*=.14; T0-T3: *P*=.07, Cohen *d*=.41. Psychological: T0-T2: *P*=.005, Cohen *d*=.58; T2-T3: *P*=.92, Cohen *d*=.20; T0-T3: *P*=.07, Cohen *d*=.36. Social: T0-T2: *P*=.02, Cohen *d*=.27; T2-T3: *P*=.67, Cohen *d*=.30; T0-T3: *P*=.32, Cohen *d*=.56. Environment: T0-T2: *P*<.001, Cohen *d*=.76; T2-T3: *P*=.49, Cohen *d*=.21; T0-T3: *P*<.001, Cohen *d*=.71.

In the follow-up evaluation (T3), both scores decreased (credibility: mean 24.34, SD 3.1; expectancy: mean 21.0, SD 4.7; Table 2). In addition, 50% (16/32) of the participants agreed and 22% (7/32) of the participants strongly agreed with the following statement: “I found it easier to address topics that were unpleasant to me in the online support group than in direct personal contact with other students.”

Repeated-measure ANOVA was performed to evaluate the differences between the scores for credibility and expectancy factors at the baseline (T0), postintervention (T2), and follow-up (T3) time points. The Mauchly test of sphericity was not assumed for either the credibility factor (Mauchly *W*=0.571; χ^2^_2_=16.8, *P*<.001) or the expectancy factor (Mauchly *W*=0.706; χ^2^_2_=10.4, *P*=.005); thus, the Greenhouse-Geisser correction was applied in both cases. Bonferroni post hoc analysis indicated that there was a statistically significant difference between the credibility factor scores. In contrast, there was no statistically significant difference in the scores between the periods of assessment for the expectancy factor (Table 2).

### Satisfaction

The postintervention (T2) evaluation showed a mean sum score of 28.66 (SD 3.02), which indicates moderately high overall satisfaction with the program. At follow-up (T3), the mean sum score was 27.91 (SD 3.02; Table 2). This outcome showed nonnormally distributed values at T2 (Shapiro-Wilk test: *P*=.002 at T2 and *P*=.10 at T3); thus, Wilcoxon tests (nonparametric paired groups) were administered. There was a statistically significant difference between the mean satisfaction scores (CSQ-8) at the postintervention (T2) and follow-up (T3) assessments (*P*=.04).

### Use of Other Support Services

The use of other support services was evaluated at the postintervention (T2) and follow-up (T3) time points. At the postintervention (T2) evaluation, 84% (27/32) of the participants indicated that they had used social contacts to help in the last 2 weeks. Furthermore, 41% (13/32) of the participants reported that they had had contact with their psychotherapist. Use of social media was reported by 47% (15/32) of the participants. WhatsApp was the most common platform (19/32, 59%), followed by Instagram (17/32, 53%) and other social media platforms (eg, YouTube and Spotify; 10/32, 31%). Other forms of support reported by 19% (6/32) of the participants were exercising, practicing yoga, meeting friends, meditating, taking pills, and reading.

At follow-up (T3), the psychological and psychiatric service provided by the university to graduate students (named “Casa 4”) was mentioned by 12% (4/32) of the participants. Again, social contacts (24/32, 75%) were the participants’ most common form of support. The number of participants reporting using social media decreased to the following: 34% (11/32) for WhatsApp, 38% (12/32) for Instagram, and 9% (2/32) for Facebook. Other forms of support reported by 19% (6/32) of the participants were exercising, practicing yoga, talking to people (face-to-face), organizing routines, and taking psychiatric medication.

### Positive and Negative Outcomes From the Online Support Groups

Participants were asked how they benefited from the online support group (Table 3). The most common answers were “I could learn from other participants’ experiences” (29/32, 91%), followed by “I felt I am not the only one facing difficulties” (28/32, 88%). In the follow-up evaluation (T3), the most common answers were “I felt I am not the only one facing difficulties” (28/32, 88%) and “I could learn from other participants’ experiences” (27/32, 84%). None of the participants reported that they had no benefit from the online support group at either time point evaluation.

**Table 3:** How students benefited from the online support groups (n=32).

Question and answer options		T2	T3
**How did you benefit as a graduate student from participating in the online support group?**			
	I felt listened to, n (%)	25 (78)	26 (81)
	I felt I am not the only one facing difficulties, n (%)	28 (88)	28 (88)
	I felt supported by other participants in the group, n (%)	20 (62)	19 (59)
	I could learn from other participants’ experiences, n (%)	29 (91)	27 (84)
	I feel more able to seek help if I need it, n (%)	12 (38)	14 (44)
	I feel more able to identify the resources I have to cope, n (%)	18 (56)	13 (41)
	I feel encouraged to take better care of myself, n (%)	22 (69)	24 (75)
	I did not benefit, n (%)	0 (0)	0 (0)
	Other, n (%)	5 (16)	3 (9)
	Other (open), participant quotes	“I felt welcomed and useful to other people, and at the end of the session, I felt more motivated to do research.” “I was able to talk to someone about my difficulties in graduate school, which I had never done before.” “I understand that I can help too.” “To have patience, respect myself and understand the pauses I need to have between one activity and another without blaming myself.” “I feel happy to be able to talk about my experiences and realize that my discourse may have helped someone.”	“It helped me to accept my own difficulties and limits.” “I feel more capable to not demand myself about what I cannot control and to accept those things I really could not do (without blaming hard, only accepting). I learned two sides of procrastination with the experiences and histories [of others].”

At the postintervention (T2) evaluation, 41% (13/32) of the participants answered the open-ended questions on negative aspects of the online support groups, and 50% (16/32) answered the questions on suggestions to improve the program. In the follow-up questionnaire (T3), 28% (9/32) and 31% (10/32) of the participants answered these questions, respectively. Overall, participants pointed out that the program could have more sessions, the sessions could last longer, and the online support groups could become a permanent university program for graduate students.

A final open-ended question asked participants to make a comment on something that they felt had not been contemplated previously. In the postintervention (T2) questionnaire, 38% (12/32) of the participants answered this field, and 28% (9/32) did so in the follow-up (T3) questionnaire. Overall, they mostly expressed gratitude for participating in the group, reported feeling very welcome, and highlighted the opportunity to share their experiences with other students. They also addressed the need to discuss mental health issues among graduate students and denaturalize constant anxiety, stress, and fear. Finally, they considered the group helpful and recognized that individual therapy might be needed to deal with more complex issues or develop topics that were not discussed in the group.

### Negative Effects

The negative effects of the intervention were evaluated at the postintervention (T2) and follow-up (T3) time points. The frequencies of negative effects, mean score, and SDs in both the postintervention (T2) and follow-up (T3) questionnaires are presented in Table 4. In the T2 assessment, 69% (22/32) of the participants scored any of the items of the NEQ, meaning that they reported having experienced at least one negative effect that might be related to the online support group. In the T3 assessment, 66% (21/32) of the participants scored any of the items of the NEQ, meaning that they reported having experienced a negative effect.

The reported negative effects were mainly related to other circumstances at both T2 (50/64, 78%) and T3 (72/85, 85%), rather than to the intervention (T2: 14/64, 23%; T3: 13/85, 15%). There were 4 missing data in T2, and 2 missing data in T3 assessments for the question of negative effects being related to the intervention or other circumstances, therefore, percentages were calculated based only on valid cases.

**Table 4:** Frequencies, means, and SDs for the Negative Effects Questionnaire (NEQ)—20 items (n=32).

Variable		T2			T3	
		Participants, n (%)	Mean (SD)	Participants, n (%)		Mean (SD)
**Frequency of negative effects (NEQ)**		68 (100)	2.1 (2.1)	87 (100)		2.7 (2.8)
	Frequency of negative effects from the intervention	14 (23)^a^	0.5 (0.8)	13 (15)^b^		0.4 (0.9)
	Frequency of negative effects from other circumstances	50 (78)^a^	1.7 (1.9)	72 (85)^b^		2.3 (2.7)
Negative impact of the intervention		N/A^c^	0.41 (1.01)	N/A		0.13 (0.34)

^a^Reduced sample size because of missing data (n=4). Percentage based on valid cases (n=64).

^b^Reduced sample size because of missing data (n=2). Percentage based on valid cases (n=85).

^c^N/A: not applicable.

At the postintervention (T2) evaluation, the most reported negative effects were “I felt more worried” (4/14, 29%) and “I think that I have developed a dependency on the online support group” (3/14, 21%). At follow-up (T3), the most frequent negative effect reported by the participants was “unpleasant memories resurfaced” (3/13, 23%).

The open question at the end of the NEQ asked participants to describe “other incidents or effects—describe in your own words whether there were any other negative incidents or effects, and what characterized them.” Participants’ answers were categorized thematically similarly to the instrument: negative effects from the intervention and negative effects from other circumstances. In the postintervention (T2) evaluation, 16% (5/32) of the participants answered it. Some situations mentioned included arguments with advisors and peers, sharing the group with a colleague from the same research group, and other external and personal situations. As an example of a negative effect of the intervention, a participant wrote the following:

On a day when everyone reported their productivity problems and bad relationship with time, I was the only one who said I managed to keep my activities normal and even became more productive in the pandemic I felt a little bad afterward...for seeming arrogant or seeming to be disdaining the group...maybe even a little isolated, I don’t know.

Female; aged 32 y; doctoral student in the second year of the course

At follow-up (T3), this question was answered by 19% (6/32) of the participants. Participants’ disclosure of their academic and professional issues and feelings of shame and anxiety were noted, whereas 9% (3/32) of the participants described no negative effects. As an example of a negative effect of the intervention, a participant wrote the following:

I felt more anxious about dealing with issues specific to my graduate program in the support group. For this memory made me feel a lump in my throat and want to cry after the meeting was over.

Female; aged 41 y; doctoral student in the fourth year of the course

### Depressive Symptoms and Quality of Life

A baseline (T0) evaluation of the PHQ-9 scores in this sample showed mild to moderate depressive symptoms (mean 9.59, SD 6.34). Nevertheless, 44% (14/32) of participants presented a sum score of ≥10, indicating clinically relevant symptoms of moderate to severe depression. Furthermore, 9% (3/32) of the participants scored ≥1 on the PHQ-9 item 9 (“thoughts that you would be better off dead, or of hurting yourself”: 0=*Not at all*, 1=*Several days*, 2=*More than half the days*, and 3=*Nearly every day*), indicating suicidality.

At the baseline evaluation (T0) of the WHOQOL-BREF for quality of life, participants had the lowest index score in the psychological domain (mean 57.03; SD 15.39) and the highest score in the social domain (mean 59.64; SD 17.21). In the postintervention (T2) evaluation, again, the psychological domain received the lowest index score (mean 64.32; SD 11.97), whereas the environment domain scored the highest (mean 68.75; SD 8.87). In the follow-up (T3) evaluation, the psychological domain had the lowest index score (mean 62.11; SD 13.15) again, and the environment domain remained with the highest score (mean 66.60; SD 11.62). Complete information on index scores for each domain of quality of life on the WHOQOL-BREF and the statistical analysis showing the differences in the scores between each assessment period (T0, T2, and T3) are presented in Table 2.

Furthermore, repeated-measure ANOVA was performed to evaluate the differences between the scores for quality of life on the WHOQOL-BREF at the baseline, postintervention, and follow-up time points. In all 4 domains, the Mauchly test of sphericity was assumed (physical domain: Mauchly *W*=0.958, χ^2^_2_=1.3, and *P*=.53; psychological domain: Mauchly *W*=1.000, χ^2^_2_=0.0, and *P*>.99; social domain: Mauchly *W*=0.994, χ^2^_2_=0.2, and *P*=.92; environment domain: Mauchly *W*=0.992, χ^2^_2_=0.2, and *P*=.89).

Overall, the scores on all domains of quality of life on the WHOQOL-BREF significantly increased from the baseline (T0) to postintervention (T2) time points (Bonferroni post hoc)—physical domain: *P*=.009, Cohen *d*=.62; psychological domain: *P*=.005, Cohen *d*=.58; social domain: *P*=.02, Cohen *d*=.27; environment domain: *P*<.001, Cohen *d*=.76. Nevertheless, the score of the environment domain was the only one that significantly differed from baseline (T0) to follow-up (T3; *P*<.001; Cohen *d*=.71). In contrast, it did not differ significantly from the postintervention (T2) to follow-up (T3) time points (*P*=.49; Cohen *d*=.21), indicating that the increase in the environment domain score observed from T0 to T2 remained in T3. The scores on the physical, psychological, and social domains did not differ significantly from the postintervention (T2) to follow-up (T3) time points (*P*>.99, *P*=.92, and *P*=.67, respectively), indicating a possible tendency for the effect to be lost over time.

## Discussion

### Sample Characteristics

This study was conducted when the COVID-19 pandemic was milder and activities at the university resumed face-to-face. Nevertheless, studies have been warning of the broad and long-lasting effects of the pandemic on students’ academic activities and mental health [68-71], highlighting the relevance of mental health promotion programs [21-28,31].

Similar to other studies conducted face-to-face [36] and over web [39,72], most of the participants were female. First, women are the majority enrolled in graduate courses in Brazil [73]. Second, the burdens on women increased during the pandemic, whereas male stigma regarding help seeking was recognized [74-76]. Interestingly, participants belonged to various faculties and, thus, represented the diversity of fields of knowledge at the university, which is outstanding for this kind of study, which usually includes psychology students [72].

The fact that more doctoral students participated in the study could be understood in light of the kind of help they need concerning the complexity of the course level. Furthermore, as most participants (23/32, 72%) were scholarship holders supported by the Brazilian government and not allowed to have other jobs, they could have more time to participate. Moreover, this may imply an income decrease, which may increase stress [12]. In fact, the financial burden was a topic often mentioned by participants during the sessions.

### Feasibility and Acceptability

The results of participants’ satisfaction, positive and negative outcomes, and effects showed the program’s feasibility and that participants were highly satisfied with it, indicating that they received the kind of support the program intended to provide [77,78]. Considering previous studies with dropout rates ranging from 7.2% to 44.2% [72,78,79], this study reported a low dropout rate of 20% (8/40). Even for the participants who missed any of the sessions, their commitment to the group could be noticed from their informing of the facilitator and justification of why they could not attend, indicating participants’ high engagement [72].

Credibility and expectancy with the online support groups were good throughout the study (baseline [T0], postintervention [T2], and follow-up [T3] time points) [80]. They might have played an important role in the participants’ satisfaction outcomes. In addition, both standardized measures and open-ended questions indicated the program’s feasibility and participants’ satisfaction at the postintervention (T2) and follow-up (T3) time points. These results may help increase the web-based intervention’s acceptability among mental health professionals [81], specifically those who work in educational contexts, contributing to the design of synchronous online support groups tailored to students.

Although many participants reported at least 1 negative effect (T2: 22/32, 69%; T3: 21/32, 66%), the frequency of negative effects was mainly related to other external circumstances (T2: 50/64, 78%; T3: 72/85, 85%; eg, arguments with advisors and peers) rather than the intervention itself (T2: 14/64, 23%; T3: 13/85, 15%)—which was overall in line with previous studies [58,59]. The rates of negative effects may vary significantly between interventions depending on the type of assessments and participants, which makes it difficult to compare negative effects across different studies [59]. Nevertheless, when it comes particularly to the negative effects of the intervention itself, the frequency in this study (T2: 14/64, 23%; T3: 13/85, 15%) was lower in comparison with the 49.9% reported in a study that used the same instrument [59] and the 93.8% reported in a study that used the Inventory for the Assessment of Negative Effects of Psychotherapy [82].

Negative effects in this study are understood as changes experienced during or after the intervention that participants considered negative and attributed to the intervention [83]. Accordingly, some adverse and unwanted events are expected to arise during psychological interventions [58,84] and may affect the outcome [85] and, therefore, should be assessed more frequently in such interventions. As research on the negative effects of psychological treatment is still not a broadly explored field [59,83,84,86], we analyzed it more exploratively while also pointing out that further studies could assess it with participants who do not necessarily have mental disorders. These results reassert the need for a crisis protocol that includes having at least one professional supporting the facilitator during the sessions who could, for example, take over in case the facilitator loses their internet connection or contact a participant to check on them if necessary. In addition, it highlights the relevance of feasibility studies in schools and educational psychology as these are more common in the field of medical and occupational research [87].

Moreover, the qualitative analysis of the suggestions and the negative aspects and effects presented by participants in the open-ended questions identified improvements regarding the program’s structure (eg, longer sessions, more sessions, smaller groups, inclusion of students from different graduate programs, and open-topic sessions), conduction (eg, more interaction among the participants), and availability (eg, becoming a continuous program offered by the university). It also showed participants’ compliance and reasserted the feasibility of the program, initially demonstrated by the standardized measures. Participants’ suggestions should be incorporated when implementing the program as they offer clues concerning its acceptability, compliance, design procedures, social validity, and practicality [87].

Furthermore, none of the participants reported that they did not benefit from the program—which is an outstanding result. First, it reasserts the advantages of web-based groups (eg, less stigmatization, ease of meeting if one feels ambivalent about attending the group, and time and money saving) [88]. Second, it supports this program’s potential benefits in promoting students’ mental health and well-being, which was the secondary outcome of this study. It points to the demand for support before students’ burdens related to the university and mental health issues are aggravated [2,5,10,12,72,89-91]. For example, a participant wrote the following:

I thought the experience was very important. The moderation approach was very careful. And, in a period when it was essential to have an experience like this to put mental health on the agenda and seek help.

Female; aged 40 y; doctoral student in the third year of the course

In addition to being feasible and highly acceptable by the participants, all the quality-of-life domains improved in the posttreatment (T2) assessment. The COVID-19 pandemic has worsened mental health indicators, quality of life, and level of physical activity among Brazilian university students [16,19,22,28,91,92] and worldwide [17,18,23-27,93]. Considering that and the fact that the topics addressed in the sessions were related to the domains measured by the WHOQOL-BREF, the overall improvement reported by participants is a remarkable outcome related to well-being and coping strategies to reduce mental distress [10]. Regarding the tendency for the effect to be lost over time observed in the physical, psychological, and social domains, we believe that it would be important to offer a longer version of the program and evaluate whether results would differ, as suggested by participants:

May it become a permanent program for graduate students, as they really are a great help.

Female; aged 44 y; doctoral student in the second year of the course

The secondary outcome of this intervention was further supported by the qualitative results, as outlined in the following section.

### Clinical Practices for Students’ Mental Health

The levels of depressive symptoms found in the baseline of this sample showed that 44% (14/32) of participants had a PHQ-9 sum score of ≥10, indicating clinically relevant symptoms of moderate to severe depression. According to Evans et al [2], graduate students are 6 times more likely to develop depression than the general population. Accordingly, the percentage of depressive symptoms in this study was higher than the 40% found in the general Brazilian population [70], whereas worldwide, a range from 14.6% to 48.3% has been reported [94]. In addition, it was higher than the results of previous studies with Brazilian university students (eg, 29% [95]), although lower than those of Brazilian undergraduate students (60.5% [22]). Internationally, university students have reported percentages of depressive symptoms ranging from 34% to 37% [21,30] (the studies did not distinguish between undergraduate and graduate students). The 9% (3/32) suicidality found in this sample could be compared with those of studies of Brazilian and international students that ranged from 7.6% to 19.6% and from 14.5% to 16.5%, respectively [21,22,25,96,97]. These results highlight the necessity and relevance of providing interventions for mental health promotion and prevention to graduate students, which has been pointed out in the literature [2,8,10,29].

The literature has pointed out precarious work conditions such as lack of regular vacation, no guarantee of labor law or legal protection while carrying out complex activities, work overload, and carrying out temporary or informal professional activities as some of the challenges faced by students [98,99]. In addition, difficulties in balancing life and studying and adapting to graduate training may be related to psychological distress and lead to physical and mental illness [98]. Relationship problems with advisors were also related to the development of depressive symptoms among graduate students [99]. In addition, students whose advisors abstained from advising presented 8% more chances of psychological distress [99]. Students with family conflicts were 52% more likely to develop psychiatric problems. Similarly, work overload increased their chance of developing mental illness by 65%. Poor sleep, fear of failure, pressure from advisors, and the culture of publishing or perishing are also issues faced by graduate students [5]. Accordingly, we noticed that the program was an opportunity for the participants to reflect on their experiences at the university and how they were coping with them. In that sense, the group functioned as a safe space to alleviate emotions, gain insights, share their issues, and receive comfort and validation [50] in addition to identifying and further developing their coping strategies.

Although this study highlighted the social validity and benefits of such interventions, it should not be considered a substitute for any other institutional initiatives that specifically address and intend to diminish structural socioeconomic inequalities (eg, racism, sexism, classism, ableism, and other discriminatory practices) presented in educational [12,100] and mental health care systems [101], which affect students’ performance and completion of studies. Nevertheless, in addition to focusing on promoting mental health, the program could be useful to screen students who need further help and refer them accordingly to university or community support services. If amplified, it could also help reduce the demand for psychological and psychiatric services in the university jointly with other mental health promotion and prevention strategies and retention initiatives.

### Implications for e–Mental Health Interventions

During the COVID-19 pandemic, many professionals had to switch their practices to a web-based format and faced many challenges to do so because of the lack of theoretical and practical knowledge or experience [81,88]. We hope that this study can contribute to the still growing literature—as studies on video groups are rare [88]—by indicating the potential benefits and negative effects of these interventions in nonclinical and subclinical populations and pointing out aspects to be considered when planning such interventions in an educational context.

A group offers an opportunity for its participants to build a mutually beneficial relationship; nevertheless, this does not mean that there will not be conflicts [102]. The facilitator plays a crucial role in mediating participants’ expectations of the group’s goals, which is an important aspect for developing group cohesion, especially over the web [81,88]. Therefore, if the facilitator does not feel comfortable, has no know-how, or has negative attitudes toward web-based interventions, this might affect the group cohesion. As web-based group interventions have their particularities compared with face-to-face interventions (eg, confidentiality, intervention frame or setting, disembodiment, and therapeutic presence) [81], it may require the facilitator to create “artificial” possibilities of overcoming some of these challenges. First, the pregroup screening interview is fundamental to state rules confidentially and instruct participants to prepare a proper environment for themselves. The absence of body-to-body communication can be diminished by verbalizing body sensations, so the facilitator could explicitly ask about body sensations [81]. As the opposite of presence is absence rather than distance [103], therapeutic presence can be achieved over web, and self-disclosure and focusing on the here and now may help achieve it [88], creating a “distanced intimacy” [103]. Accordingly, specific training is recommended to increase the facilitator’s self-confidence and for them to learn how to develop a therapeutic alliance and group cohesion over web [81]. Finally, as the opportunities for interacting beyond the group session are limited (unlike those that could happen spontaneously in face-to-face interactions, such as small talks before and after the sessions and exchange of contact information), the facilitator may offer some asynchronous channels for participants to interact if they want to.

For ethical reasons, participants should be informed of the potential negative effects of taking part in similar interventions. For example, school-based programs may adapt consent forms similar to those used in research study protocols for students to sign to participate in the intervention, as well as discussing it in the pregroup meeting. As stated previously, having a crisis protocol is important, and it must be adapted to the support system that is available (eg, having information on the participant’s contact person in case of emergency and health and emergency services that are close to the participant’s location).

Given the high participation rate in the intervention, we hope that this study inspires universities to support their students with low-threshold interventions such as this one combining them with other psychosocial support services.

### Strengths and Limitations

This study has some limitations. First, the sample size was relatively small, although it was similar to those reported in previous feasibility studies. Second, the negative effects of psychological interventions or treatments need a construct consensus and standardization of measures [59,83,84]. The overall results (eg, the total number of negative effects reported or the mean negative impact) we present are limited to interpretation or comparison with other studies; thus, they should be considered exploratory variables. Second, the program was designed and took place during the COVID-19 pandemic, and this must be considered when evaluating the results we presented. Fourth, the intervention was designed as a mental health promotion strategy and not primarily to have a specific effect on depressive symptoms, so we did not assess depressive symptoms at T2 and T3. A further study could focus on evaluating the effectiveness of the intervention on depressive symptoms (eg, in a population presenting clinically relevant depressive symptoms).

Nevertheless, it is—to our knowledge—the first study in Brazil to evaluate whether an online support group program was feasible and acceptable, and the results showed that it was feasible and highly acceptable among the participants. On the basis of participants’ commitment to the group and their feedback on the program, we considered their compliance to be very good. For example, further studies could compare the program’s web-based and face-to-face formats. Moreover, important steps for future studies could be to adapt the intervention design based on participants’ feedback, conduct a randomized controlled trial performing a cost-effectiveness analysis, and assess the intervention’s effect on rates of discontinuation of studies.

### Conclusions

Online support groups for mental health promotion for graduate students are feasible, usable, and easily implemented in the university context. They are also satisfactory and may positively influence different domains of participants’ quality of life. Therefore, they can be considered a helpful mental health promotion strategy in the educational context independent of social distancing measures. Considering its characteristics (online, group format, and number of sessions), we may also claim its good cost-effectiveness, especially for public universities in low- and middle-income countries that may have students allocated to different cities. Further studies could evaluate these (or similar) programs under nonpandemic circumstances.

## Abbreviations

CEQ: Credibility and Expectancy Questionnaire
CSQ-8: Client Satisfaction Questionnaire–8
NEQ: Negative Effects Questionnaire
PFA: Psychological First Aid
PHQ-9: Patient Health Questionnaire–9
UFPR: Federal University of Paraná
WHOQOL-BREF: abbreviated World Health Organization Quality of Life assessment

## Data Availability

The data of this study cannot be made publicly available because of ethical restrictions imposed by the Brazilian National Health Council, represented by the Federal University of Paraná ethics committee in Brazil. However, the data supporting this study’s findings are available from the corresponding author upon reasonable request.
